# Evaluation of and implications for a novel hepatitis C e-consult direct-to-treatment pilot program

**DOI:** 10.1038/s41598-023-43052-7

**Published:** 2023-10-11

**Authors:** Neaka Z. Mohtashemi, Crystal Y. Teng, Jihane Benhammou, Tien Dong, Matthew Bidwell Goetz, Arpan Patel, Jenna Kawamoto, Debika Bhattacharya

**Affiliations:** 1 Veteran Affairs Greater Los Angeles Healthcare System, Los Angeles, CA USA; 2 David Geffen School of Medicine at the University of California Los Angeles, Los Angeles, CA USA

**Keywords:** Viral hepatitis, Health care

## Abstract

A Hepatitis C (HCV) e-Consult Direct-To-Treatment (DTT) program managed by midlevel providers was developed at the Veteran Affairs Greater Los Angeles Healthcare System (VAGLAHS) which provided remote referral and, in some, remote management of HCV. DTT patients were more likely to be initiated on HCV treatment compared to standard of care (SOC), lending support for similar programs of remote engagement in HCV care.

## Introduction

An estimated 2.4 million people in the United States have chronic hepatitis C virus (HCV) infection^1^, with Veterans having a higher rate (5.4%) than the general U.S. population (1.8%)^2^. Treatment is recommended for all persons living with HCV, with life expectancy > 12 months^3^. The Department of Veteran Affairs (VA) has been a leader in hepatitis treatment, particularly with the advent of Direct-Acting Antivirals (DAAs) in 2014^4^. The pillars of the VA’s elimination initiative are novel HCV screening and treatment programs, including mid-level provider care management and telehealth^4–6^. Furthermore, the COVID-19 pandemic has impacted HCV screening and treatment, emphasizing the increased need for telehealth interventions for HCV and generally^7^. Hepatitis C virus antibody testing decreased by 59% in April 2020 and hepatitis C virus RNA-positive results fell by 62% in March 2020, with continued reductions by 39% in July 2020, while hepatitis C virus treatment prescriptions decreased by 43% in May, 37% in June, and 38% in July compared to corresponding months in 2018 and 2019, highlighting the importance of novel methods of screening, linkage to care, and treatment^8^.

Indeed, one of the barriers to HCV evaluation and treatment may be timely access to HCV providers. Studies have outlined difficulty with specialist referrals and long specialist wait times as barriers to HCV treatment^9–11^. Quickly identifying the appropriate HCV provider and limiting confusion for the person living with HCV is an important component of effective treatment^9^. At VAGLAHS, a hepatitis C e-Consult Direct-To-Treatment (DTT) program was established from March 2016 to November 2017 to expedite treatment evaluation. In this pilot program, primary care providers were offered an option to select an “e-consult” for HCV management. A midlevel practitioner reviewed the electronic medical record to determine treatment eligibility within 3 business days and used an algorithm to determine HCV treatment eligibility. If deemed eligible, the midlevel practitioner contacted the Veteran and directly initiated HCV therapy. If deemed ineligible, the Veteran was directed to evaluation by the infectious diseases/hepatology service. Standard of care was defined as treatment evaluation through the usual referral pathway, which required an initial face to face visit in the infectious diseases/hepatology clinic. This program operated from March 2016–November 2017.

As we evaluate ways to improve HCV care delivery in the DAA and pandemic era, we sought to characterize this historical pilot program of Veterans that were referred to the HCV e-consult DTT program, and to compare the health care utilization and clinical outcomes of patients who initiated HCV treatment via DTT versus referral via SOC practices.

## Methods

**Figure 1:**
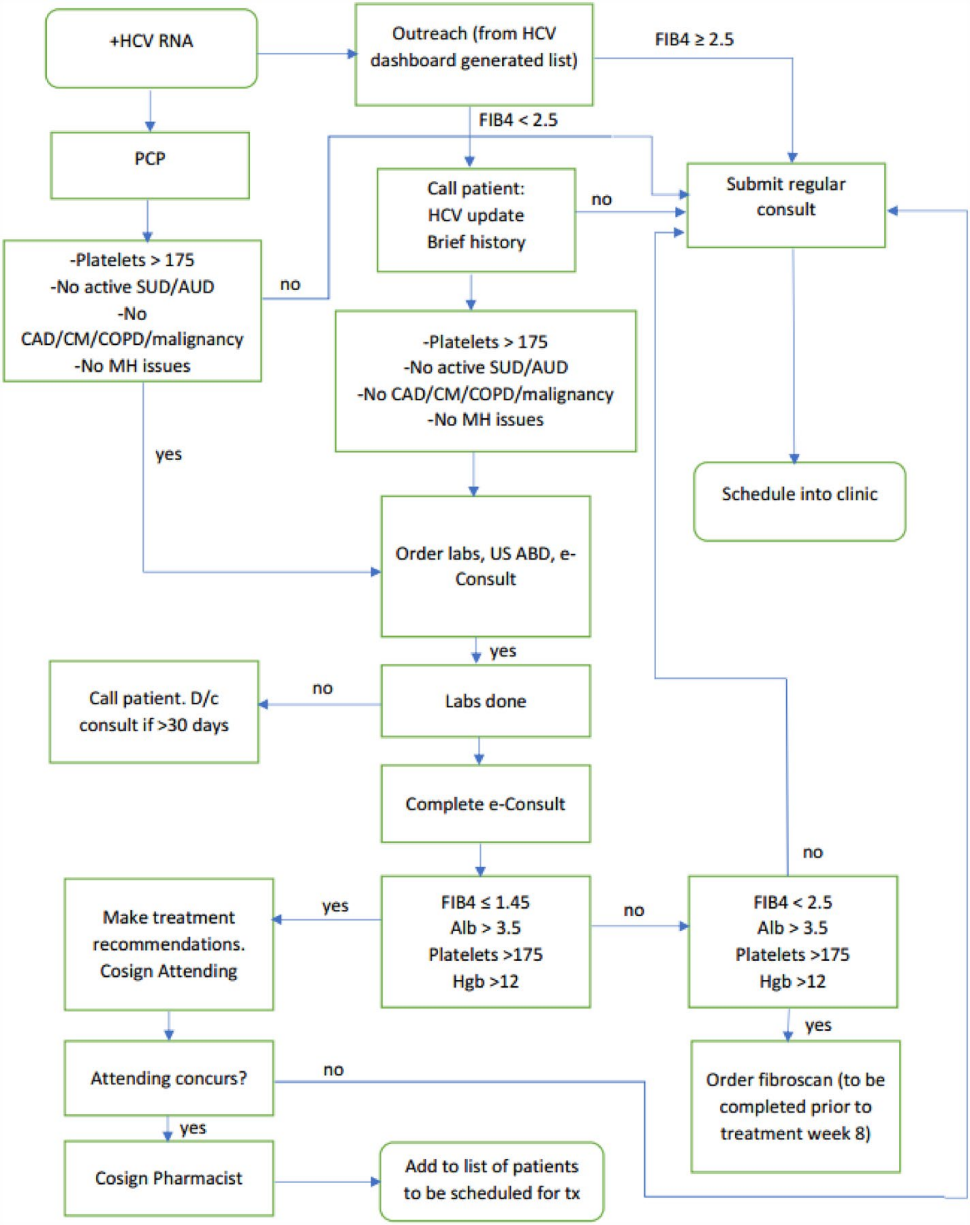
HCV e-consult process.

This retrospective chart review study was conducted at the VAGLAHS to evaluate the HCV e-consult mechanism (Fig. 1). Adults 18 years or older who had an HCV e-consult submitted between March 1, 2016 and November 13, 2017 were included.

Data was collected via chart review from VAGLAHS using the Computerized Patient Record System. Demographic information (age, gender, race, ethnicity, social history and medical history) was collected at the time of HCV e-consult evaluation. Treatment eligibility criteria are highlighted in Supplemental Table 1. Briefly, patients without clinical cirrhosis, fibrosis, substance abuse disorder, alcohol use disorder, malignancy, mental health disorders, chronic obstructive pulmonary disease, or heart disease were eligible for inclusion. Endpoints included HCV treatment initiation (yes/no), HCV treatment completion (yes/no), SVR12 (defined by ≥ 10 weeks post-treatment), and the number of HCV treatment-related clinic visits. Outcomes were compared between Veterans who initiated treatment via DTT and patients who were ineligible for DTT and referred to standard of care. Descriptive statistics, t-tests, and chi-squared tests were used.

**Table 1:** Demographics and clinical outcomes.

	Eligible for DTT N = 85	Ineligible for DTT N = 101	*p*-value
Age (years), mean ± SD	60.8 ± 8.0	63.1 ± 8.6	0.066
Gender, n (%)			
Male	83 (98%)	99 (98%)	0.861
Race, n (%)			
American Indian or Alaska Native	2 (2%)	3 (3%)	
African American	31 (36%)	47 (47%)	
Pacific Islander	2 (2%)	0 (0%)	
White	35 (41%)	41 (41%)	
Ethnicity, n (%)			
Hispanic/Latino	10 (12%)	13 (13%)	0.819
Social history			
Alcohol use^†^, n (%)			0.575
Within past 2 years	10 (12%)	10 (10%)	
History (> 2 years)	18 (21%)	28 (28%)	
Drug use^†^, n (%)			0.090
Within past 2 years	8 (9%)	19 (19%)	
History (> 2 years)	22 (26%)	31 (31%)	
Polysubstance abuse^†^, n (%)			0.085
Within past 2 years	1 (1%)	8 (8%)	
History (> 2 years)	6 (7%)	9 (9%)	
Homelessness, n (%)			0.699
Current or within past 2 years	5 (6%)	19 (19%)	
History (> 2 years)	9 (11%)	13 (13%)	
Medical history, n (%)			
Coronary artery disease	3 (4%)	7 (7%)	0.306
COPD	9 (11%)	11 (11%)	0.947
Congestive heart failure	0 (0%)	1 (1%)	1.000
Malignancy	3 (4%)	12 (12%)	0.037
Mental health disorder	41 (48%)	52 (51%)	0.659
HCV genotype, n (%)			0.111
1a	47 (55%)	51 (50%)	
1b	20 (24%)	22 (22%)	
2	12 (14%)	9 (9%)	
3	3 (4%)	13 (13%)	
4	0 (0%)	1 (1%)	
Prior HCV treatment status, n (%)			
Treatment naïve	77 (91%)	92 (91%)	0.906
Liver fibrosis scores, mean ± SD			
FIB-4	1.44 ± 0.42	2.62 ± 1.83	< 0.001
APRI	0.41 ± 0.19	0.79 ± 0.72	< 0.001
Advanced fibrosis (FIB4 > 3.25), n (%)	0 (0%)	21 (21%)	< 0.001
DAA treatment initiation			
Initiated DAA therapy, n (%)	60 (71%)	56 (55%)	0.034
Days to treatment initiation, mean ± SD	90.4 ± 66.9	106.4 ± 69.4	0.210
Median days to treatment initiation (range)	68.5 (12–294)	84.5 (20–295)	
Treatment status*, n (%)			
Completed treatment	45 (75%)	43 (77%)	0.822
Discontinued treatment	5 (8%)	3 (5%)	0.527
DAA regimen, n (%)			
Sofosbuvir/ledipasvir	42 (70%)	40 (71%)	
Sofosbuvir/velpatasvir	15 (25%)	10 (18%)	
Elbasvir/grazoprevir	2 (3%)	2 (4%)	
Glecaprevir/pibrentasvir	1 (2%)	0 (0%)	
Sofosbuvir/ledipasvir + ribavirin	0 (0%)	1 (2%)	
Sofosbuvir/velpatasvir + ribavirin	0 (0%)	2 (4%)	
Sofosbuvir/velpatasvir/voxilaprevir	0 (0%)	1 (2%)	
SVR12, n (%)			
SVR12 achieved**	19 (95%)	17 (94%)	0.939
Adverse events, n (%)	10 (17%)	6 (11%)	0.353
Follow-up visits, mean ± SD			
Number of HCV clinic follow-up visits	3.00 ± 0.73	4.20 ± 1.27	< 0.001

*Patients that are not represented are treatment in progress. N = 10 (17%) and N = 10 (18%) for eligible and non-eligible patients, respectively.

**Based on patients who completed SVR12 labs. N = 20 (61%) and N = 18 (69%) for eligible and non-eligible patients, respectively.

^†^Any history of alcohol use, substance use, or polysubstance use; only those with non-active previous use were eligible for DTT.

Achievement of SVR12 was based upon patients who completed SVR12 labs. Of those who completed treatment but did not reach SVR12, one patient had virologic failure, while 24 missed their lab collection. Adverse events were reported common events, evaluated based on the patients’ HCV clinical treatment notes. These included headache, nausea, fatigue, diarrhea, weight loss, insomnia, suppressed appetite, and itchy redness and burning on lower extremities. Only one person discontinued HCV treatment due to adverse events.

### Ethics approval and consent to participate

The study was approved by the local institutional review board (IRB) at VAGLAHS, and all study activities were carried out in accordance with the relevant guidelines. Informed consent was waived by the VAGLAHS IRB due to the retrospective nature of this investigation.

## Results

One hundred and ninety-four (194) HCV e-consults were completed during the study timeframe. Seven patients with undetectable viral loads and 2 duplicate e-consults were excluded. Of 186 e-consults, 85 patients (46%) were eligible to initiate HCV treatment via DTT. Veterans were predominantly male (98%) with an average age of 61 ± 8 years and 63 ± 9 years for eligible and non-eligible patients, respectively (*p* = 0.066) (Table 1). Compared to those not eligible for DTT, DTT eligible patients had lower rates of recent drug use (9% vs 19%) and history of drug use (26% vs 31%) (*p* = 0.090). Similarly, eligible patients had lower rates of recent polysubstance use (1% vs. 8%, *p* = 0.085). Baseline comorbidities were similar among both groups, except for malignancy, which was lower in DTT eligible patients (4% vs 12%, *p* = 0.037). HCV genotype was similarly distributed between the two groups, apart from genotype 3 which was less frequent in DTT eligible patients (4% vs. 13%). Eligible patients also had lower FIB-4 (1.44 ± 0.42 vs. 2.62 ± 1.83, *p* < 0.001) and APRI scores (0.41 ± 0.19 vs. 0.79 ± 0.72, *p* < 0.001), and none of the eligible patients had advanced fibrosis as compared to non-eligible patients (0% vs 21%, *p* < 0.001).

More Veterans received treatment when initiated through the DTT program as opposed to those who received SOC (71% vs 55%, *p* = 0.034) and the DTT group also had fewer mean face-to-face clinic visits during HCV treatment (3.0 ± 0.7 vs 4.2 ± 1.3, *p* < 0.001). Of patients starting treatment (DTT vs SOC), the mean time to HCV treatment initiation (90.4 ± 66.9 vs 106.4 ± 69.4 days, *p* = 0.210) and treatment completion (75% vs 77%, *p* = 0.822) were similar. Importantly SVR12 rates were similar (95% vs 94%, *p* = 0.939). Rates of adverse events were also comparable for DTT vs SOC (17% vs 11%, *p* = 0.353) (Table 1).

## Discussion

This retrospective chart review evaluated a historical HCV e-consult DTT program with the aim of comparing clinical outcomes of patients who initiated HCV treatment via DTT versus SOC practices. Veterans eligible for DTT were more likely to initiate DAA therapy than those who were not. There was also a trend towards shorter time to HCV treatment initiation in patients who initiated via DTT as compared to ineligible patients who were referred to specialists. HCV treatment completion and SVR12 rates were similar for both groups, but the DTT group had fewer face-to-face clinic visits during HCV treatment.

To our knowledge, this is the first evaluation of a program which referred patients directly to a remote online evaluation system (HCV e-consult DTT program) with subsequent successful HCV treatment initiation and completion by midlevel providers, highlighting a potential model for other integrated healthcare systems. There exist other telehealth programs that have been demonstrated to be effective for HCV treatment^12–15^. Of particular note, at the VA, one study found that sites that offered video conferencing between primary care providers and people living with HCV as part of the HCV VA-ECHO program had higher treatment initiation rates than sites that did not offer this^16^. However, this program and others did not utilize the same e-consult DTT model reported here. The novelty of the e-consult DTT program lies in the potential for reduced time between HCV diagnosis and treatment. Furthermore, this DTT program has implications for pandemic-era losses in screening, linkage to care, and treatment initiation. The COVID-19 pandemic saw reduced HCV screening and treatment across all populations; telehealth interventions can close this gap by offering safer, more convenient healthcare alternatives for people affected by the pandemic.

We report SVR12 rates of 95% with this program. Other programs that have implemented remote linkage to care and/or treatment have found similar success, with SVR rates between 93.3–98.5%^17–19^. However, these programs differed from the model described here in a few important ways. Linkage to care and treatment were either not managed or not exclusively managed by midlevel practitioners^17–19^, they did not take place in integrated healthcare systems^18,19^, and some exclusively evaluated people who inject drugs (PWID)^18,19^.

This cohort was evaluated 7 years ago; as such, there have been advances in telehealth outreach and technology since. Moving forward, these data are important due to their demonstration of the effectiveness of telehealth and DTT interventions in a time when these programs were less prevalent. The success of the program in linking Veterans to care and achieving SVR underlines the utility of telehealth programs with midlevel providers, particularly as specialists are often less accessible. Additionally, it is important to note that, now that telehealth programs are more established, those with substance abuse and alcohol abuse disorders are more likely to be given the opportunity to be treated via electronic means.

Limitations include a limited sample size and short study timeframe. Second, this study was completed at a single healthcare facility with Veterans; thus, results may not be applicable to other populations. The study also did not evaluate patient acceptance of the program or include any quality of life indices. Those who received treatment via SOC were likely a disparate population compared to those referred through DTT. With regards to the higher proportion of DTT patients initiating therapy, it is possible that those referred to standard of care had more comorbidities, requiring more diagnostic evaluation that delayed care. Future research could evaluate clinical outcomes in patients with similar baseline characteristics who initiated treatment via DTT versus SOC.

As compared to SOC, the HCV e-consult DTT program managed by midlevel practitioners required fewer visits for patients to achieve similar SVR12 rates, results which may have important cost-saving and post-pandemic-era safety ramifications. Overall, our findings lend support for this e-consult program in other integrated healthcare systems.

### Supplementary Information

Supplementary Information.

## Data Availability

The data analyzed during this study are not available due to the sensitive nature of health information.
