# Supplementary material for: Evaluation of and implications for a novel hepatitis C e-consult direct-to-treatment pilot program
Source: Sci Rep. 2023 Oct 11;13:17241. doi: 10.1038/s41598-023-43052-7 (PMC10567689; doi:10.1038/s41598-023-43052-7)
Supplement: Supplementary file 1 — Supplementary Information. [file 41598_2023_43052_MOESM1_ESM.docx]

**Supplementary Table 1. HCV E-Consult Eligibility Criteria**

| HCV E-Consult Eligibility Criteria* |
| --- |
| Lab values |
| FIB4 <2.5 |
| Albumin >3.5 |
| Platelets >175 |
| Hemoglobin >12 |
| Imaging |
| Abdominal ultrasound to be completed before (preferred) or during treatment |
| FibroScan prior to treatment week 8 required for patients with FIB-4 between 1.45 and 2.5 |
| No active or uncontrolled comorbidities (such as the following): |
| Substance use disorder |
| Alcohol use disorder |
| Coronary artery disease |
| Cardiomyopathy |
| Chronic obstructive pulmonary disease |
| Malignancy |
| Mental health disorders |

*Patients with HCV genotype 3 used to be excluded from the e-consult

**Supplementary Table 2. Reasons for Ineligible Hepatitis C e-Consults**

| Reasons for Ineligible E-Consults | Number of Subjects |
| --- | --- |
| FIB4 ≥2.5 | 35 (35%) |
| Platelets ≤175 | 31 (31%) |
| Evidence of cirrhosis | 15 (15%) |
| HCV Genotype 3 | 13 (13%) |
| Hemoglobin ≤12 | 13 (13%) |
| Active substance use | 11 (11%) |
| Mental health disorder | 8 (8%) |
| Malignancy | 6 (6%) |
| Could not reach patient | 4 (4%) |
| Chronic obstructive pulmonary disease | 2 (2%) |
| Coronary artery disease | 2 (2%) |

*Denominator was the total number of patients ineligible for DTT (n=101). Patients often had more than one reason for not being eligible.
